# The BH3 mimetic (±) gossypol induces ROS-independent apoptosis and mitochondrial dysfunction in human A375 melanoma cells in vitro

**DOI:** 10.1007/s00204-021-02987-4

**Published:** 2021-02-01

**Authors:** Lisa Haasler, Arun Kumar Kondadi, Thanos Tsigaras, Claudia von Montfort, Peter Graf, Wilhelm Stahl, Peter Brenneisen

**Affiliations:** Institute of Biochemistry and Molecular Biology I, Medical Faculty, Heinrich Heine University Düsseldorf, Düsseldorf, Germany

**Keywords:** Malignant melanoma, Gossypol, Mitochondrial dysfunction, Apoptosis, ROS

## Abstract

A major challenge in current cancer therapy is still the treatment of metastatic melanomas of the skin. BH3 mimetics represent a novel group of substances inducing apoptosis. In this study, we investigated the cytotoxic effect of (±) gossypol (GP), a natural compound from cotton seed, on A375 melanoma cells and the underlying biochemical mechanisms. To prevent undesired side effects due to toxicity on normal (healthy) cells, concentrations only toxic for tumor cells have been elaborated. Viability assays were performed to determine the cytotoxicity of GP in A375 melanoma and normal (healthy) cells. For the majority of experiments, a concentration of 2.5 µM GP was used resulting in a ROS-independent but caspase-dependent cell death of A375 melanoma cells. At this level, GP was non-toxic for normal human epidermal melanocytes. GP has a very short half-life, however, it was demonstrated that only the “parent” compound and not decomposition products are responsible for the cytotoxic effect in A375 melanoma cells. GP significantly decreased mitochondrial membrane potential accompanied by a Drp1-dependent loss of mitochondrial integrity (fragmentation) in tumor cells. Taken together, GP induced a ROS-independent intrinsic apoptosis leading to the conclusion that within a specific concentration range, GP may work as effective anticancer drug without harmful side effects.

**Supplementary Information:**

The online version contains supplementary material available at 10.1007/s00204-021-02987-4.

## Introduction

Malignant melanoma is the most aggressive type of skin cancer due to its high potential to metastasize resulting in poor survival (Janostiak et al. 2019; Miller and Mihm 2006; Zbytek et al. 2008). After discovery of B-RAF as an oncogene in some cancer types and of V600E being the most relevant somatic missense mutation on B-RAF in melanoma (Davies et al. 2002; Wellbrock et al. 2004), specific B-RAF inhibitors were applied to treat melanoma and improved treatment effects (e.g. increased response rate) were observed compared to conventional chemotherapeutic agents (Chapman et al. 2011; Flaherty et al. 2012). However, the response rate was only about 50% and patients still develop resistance within several months (Flaherty et al. 2012; Nazarian et al. 2010). In addition, immunotherapy is used to treat melanoma, but this option is limited because not all patients respond to this therapy. In addition, severe side effects such as susceptibility to autoimmune diseases were observed (Larkin et al. 2015; Luke et al. 2017). Increasing resistance requires the development of new approaches to effectively treat these types of cancer. One focus is on stimulation of the programmed cell death, apoptosis (Mohammad et al. 2015), that is often inhibited in cancer cells (Hanahan and Weinberg 2011). Key regulators in this pathway are members of the Bcl-2 protein family which is classified into anti- (e.g. Bcl-2, Bcl-xL, and MCL-1) and pro-apoptotic (e.g. Bax, Bak, Bim, and Bad) proteins, related to function and number of homology domains (BH1–4) (Cory and Adams 2002; Opydo-Chanek et al. 2017; Vogler 2014). During cellular homeostasis, there is a functional balance between these groups of molecules. However, a shift to more anti-apoptotic proteins leads to survival and pathological alteration in cancer cells (Korsmeyer et al. 1993; Mohammad et al. 2015). Several tumor cells including melanoma cells (Lee et al. 2019) show an overexpression of pro-survival proteins, thus escaping apoptosis and becoming resistant against pro-apoptotic signaling (Vogler 2014; Yip and Reed 2008; Zhang et al. 2002).

**Fig. 1:**
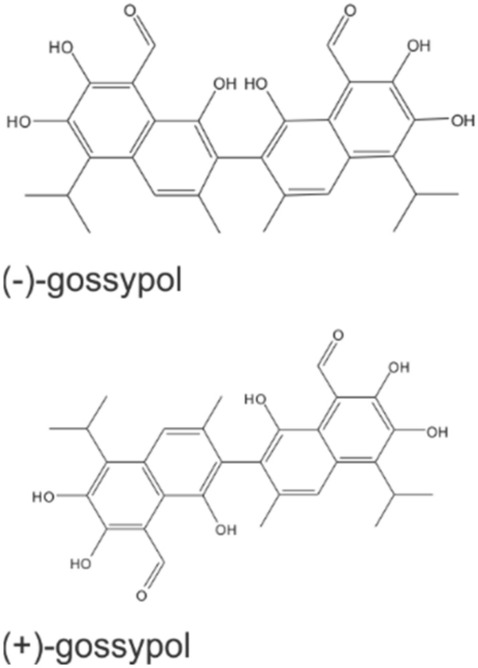
Chemical structure of GP enantiomers (+, −)

To overcome the resistance, small molecules, so called BH3 mimetics, have been developed to antagonize the anti-apoptotic proteins leading to the induction of the intrinsic pathway of apoptosis (Mohammad et al. 2015; Zhang et al. 2007). BH3 mimetics can be grouped into peptides and non-peptide synthetic small molecules, with the latter already tested in clinical trials (Opydo-Chanek et al. 2017). For example, ABT-737, an acrylsulfonamide-based pan Bcl-2 inhibitor that binds to Bcl-xL, Bcl-2 and Bcl-w, was shown to exert potent single-agent activity in leukemia and lymphoma cells lines as well as synergistic effect in combination with other anticancer drugs in leukemia and solid tumors (High et al. 2010; Konopleva et al. 2006; Oakes et al. 2012). However, this compound has a poor bioavailability, and thus, new derivatives have been explored (Lin et al. 2017; Park et al. 2008; Tse et al. 2008). Gossypol (GP), a natural polyphenolic aldehyde (Fig. 1) found in cotton seed plants (Adams et al. 1960), was originally developed as an oral contraceptive for men (Wu 1989), but is now also being tested for use as an anticancer drug (Opydo-Chanek et al. 2017; Zeng et al. 2019). GP binds to Bcl-xL, Bcl-2, MCL-1 and Bcl-w and thus, act as a pan Bcl-2 inhibitor (Kang and Reynolds 2009; Kitada et al. 2003; Lessene et al. 2008; Oliver et al. 2005). Natural GP contains (+) and (−) enantiomers, the latter named AT-101 is readily bioavailable after oral application and exhibits suitable pharmacokinetic properties (Opydo-Chanek et al. 2017). In this context, Janostiak and coworkers showed that both B-RAF kinase inhibitor (Vemurafenib) plus anaplastic lymphoma kinase (ALK) inhibitor (Ceritinib) resistant A375 melanoma cells isolated from xenograft mice are sensitive to AT-101 treatment. At concentrations ≥ 5 µM of AT-101, the relative cell survival was ≤ 50% after 72 h (Janostiak et al. 2019). However, the underlying mechanism and the effect of those concentrations on normal (healthy) cells were not studied. In this study, we focused on the mode of action of GP in A375 melanoma cells in comparison to normal melanocytes.

## Materials and methods

### Materials

All chemicals were purchased from Sigma-Aldrich (Taufkirchen, Germany) or Merck (Darmstadt, Germany), unless otherwise stated. ( ±) Gossypol (GP, CAS 303-45-7) was obtained from Abcam (Cambridge, UK). The purity of GP was > 98% according to the manufacturer’s specifications. DMSO and TRIS were from Roth (Karlsruhe, Germany). The fetal bovine serum (FBS) was from Pan-Biotech (Aidenbach, Germany). Penicillin/Streptomycin was purchased from Biochrom and Glutamax from Gibco (Darmstadt, Germany). The melanocyte growth medium (C-24019), the growth medium supplement mix (C-39415) and the melanocyte basal medium (C-24210) were obtained from PromoCell (Heidelberg, Germany). Z-VAD(OMe)-FMK was purchased from Santa Cruz Biotechnology (Heidelberg, Germany). Molecular Probes MitoTRACKER™ Green and tetramethylrhodamine methyl ester (TMRM) were obtained from Thermo Fisher Scientific (Waltham, Massachusetts, USA). Hank’s Balanced Salt Solution (HBSS) was from Gibco (Darmstadt, Germany). Primary antibodies were used: anti-PARP (9542), anti-Smac/DIABLO (15108), anti-Bax (5023), anti-Drp1 (14647) and beta-actin from Cell Signaling Technology (Massachusetts, USA), anti-caspase 3 (ab32351) from Abcam (Cambridge, UK), anti-TOM20 from Proteintech (St. Leon-Rot, Germany), anti-GAPDH (G8795) from Sigma-Aldrich and anti-Tim23 (611222) from BD Transduction Laboratories (Bath, UK); secondary antibodies were: horseradish peroxidase (HRP)-conjugated goat anti-rabbit IgG (111–035-144) from Dianova (Hamburg, Germany) and HRP-conjugated rabbit anti-mouse IgG from Dako (Glostrup, Denmark), DC™ protein assay kit was purchased from Bio-Rad (Feldkirchen, Germany).

### Cell culture

Human melanoma cell line A375 (ATCC^®^ CRL-1619) was obtained from the American Type Culture Collection (ATCC, Virginia, USA). Normal human epidermal melanocytes (NHEM, C-12400) were from PromoCell (Heidelberg, Germany). Melanoma cells were cultured in Dulbecco’s Modified Eagle’s (DMEM, low glucose), supplemented with 10% fetal bovine serum (FBS), streptomycin (100 µg/ml), penicillin (100 U/ml) and GlutaMAX™ (2 mM) at 37 °C in 5% CO_2_. Melanocytes were cultured in melanocyte growth medium, supplemented with growth medium supplement mix, streptomycin (100 µg/ml) and penicillin (100 U/ml) at 37 °C in 5% CO_2_. Subconfluent cells (70–80% confluence) were used for all experiments. A375 cells were incubated in high glucose (4500 mg/L) DMEM without FBS and NHEM in melanocyte basal medium. The substances were directly added to the cells.

### Cell viability assays

*MTT assay*, cell viability was measured by the MTT (3-(4,5-dimethylthiazol-2-yl)-2,5-diphenyltetrazolium bromide) assay which is based on the activity of mitochondrial dehydrogenase (Mosmann 1983). Briefly, the enzyme catalyzes the conversion from MTT to a purple formazan dye which can be measured by absorbance. Subconfluent cells were treated with different concentrations of GP or 2 mM H_2_O_2_ as positive control in 24-well plates. After washing with PBS, MTT solution (0.5 mg/ml) was directly added to the cells which were incubated between 0.5 and 2 h depending on the cell line. Subsequently, MTT was removed, the cells were washed once with PBS and 0.5 ml DMSO was added for formazan extraction. Absorbance was measured at 570 nm with a plate reader (FLUOstar OPTIMA, BMG Labtech, Ortenberg, Germany). The mock-treated control was set at 100%.

*SRB assay*, cell viability was measured using the dye Sulforhodamine B (SRB) (Maydt et al. 2013). The principle is based on the pH-dependent staining of total proteins with SRB. The assay was performed with subconfluent cells in 24-well plates. Cells were treated with different concentration of GP or 2 mM H_2_O_2_ (positive control). Subsequently, cells were washed with PBS and fixed with 0.5 ml 10% (w/v) cold trichloroacetic acid solution for 1 h at 4 °C. After washing five times with dH_2_O, cells were dried at RT. For the staining, cells were incubated with 0.3 ml SRB solution (0.4% (w/v) in 1% acetic acid) for 15 min at RT, washed 5 × with 1% acetic acid and dried at RT again. For extraction of SRB, 0.4 ml TRIS-Base (10 mmol/l) was added per well and the plate was gently rotated for 5 min. The absorbance was measured at 492 nm minus background values at 620 nm using a plate reader (Tecan M200 pro, Männedorf, Switzerland). Cell viability of control cells was set at 100%.

### Extra- and intracellular measurement of GP (HPLC)

Cellular uptake of GP was determined by HPLC after treatment of cells with 5 µM GP. Cells were grown to subconfluence in Ø 10 cm culture dishes. After incubation of GP for different time periods, cells were washed with PBS twice and harvested in 2 ml PBS. Samples were centrifuged at 500×*g* for 8 min at 4 °C, washed with PBS and centrifuged at 5000×*g* for 6 min at 4 °C. Cell pellets were resuspended in 150 µl acetonitrile (AcN), mixed thoroughly and centrifuged at 20,000×*g* for 5 min at 4 °C. For HPLC analysis, 50 µl of the AcN extract was injected, and the GP concentration was quantified using a standard curve. HPLC was performed on a Supelco pKb 100 (250 × 4.6 mm) column with a mobile phase consisting of AcN/water/trifluoroacetic acid (90/10/0.1, v/v/v) at a flow rate of 1.0 ml/min (0–10 min) and detection at 367 nm. Retention time of GP was around 5 min. In this approach, we measured only GP and not its metabolites in the cells. For protein quantification, the solvent residue was evaporated and the cell pellet was solved in 1% SDS lysis buffer containing 0.1% protease inhibitor cocktail and sonicated. Protein concentrations were determined with the DC™ Protein Assay Kit. For quantification of the intracellular GP content, the concentration of GP was set in relation to the protein amount. For determination of the detectable amount of GP (*t* = 15 min) in the cells, we calculated the quotient of the total amount of GP measured within the cells and the total amount added extracellularly, normalized to the protein amount. The mean value of the detected amount in all cells was calculated.

### Mitochondrial membrane potential (*ΔΨ*_*m*_)

To monitor the Δ*Ψ*_*m*_, melanoma cells or melanocytes were seeded on glass bottom dishes (Ø 3.5 cm, MatTek, Son, Netherlands) and incubated with 2.5 µM GP for 2 and 4 h, respectively, or with 10 µM carbonyl cyanide m-chlorophenyl hydrazine (CCCP), an oxidative phosphorylation uncoupler (Leblanc 1971), for 2 h as positive control. After incubation, cells were washed with PBS once and loaded with 100 nM of the mitochondrial membrane potential sensitive dye tetramethylrhodamine methyl ester (TMRM) and 100 nM of the mitochondrial membrane potential insensitive dye MitoTRACKER™ Green for 0.5 h at 37 °C. The accumulation of the fluorescent TMRM in intact mitochondria leads to a bright fluorescence signal which is weakened by adding apoptotic or other stressors, indicating a loss of mitochondrial membrane potential (Creed and McKenzie 2019; Scaduto and Grotyohann 1999). Subsequently, cells were washed with PBS once and fresh medium was added to the cells. Cells were analyzed with a spinning disc confocal microscope (PerkinElmer, Waltham, Massachusetts, USA). At least 20 image stacks per sample were evaluated. The Δ*Ψ*_*m*_ is calculated by TMRM/MitoTRACKER™ Green quotient and the mock-treated control was set at 100%.

### Mitochondrial fragmentation

To test for mitochondrial fragmentation, melanoma cells and melanocytes were treated as described above for Δ*Ψ*_*m*_. MitoTRACKER™ Green images were used to evaluate mitochondrial morphology, quantification was performed as described before (Duvezin-Caubet et al. 2006): tubular, at least one mitochondrial tubule of 5 µm or more; intermediate, at least one tubule between 0.5 and 5 µm; fragmented, no tubules of more than 0.5 µm in length. At least 30 cells per sample were analyzed.

### Fluorescent measurement of H_2_O_2_ using Hyper and SypHer

Human melanoma A375 cells were transfected using GeneJuice (Merck) with 1 µg of one of the following plasmids: Hyper (cytosolic), SypHer (cytosolic), mito-Hyper (mitochondrial matrix targeted) and mito-SypHer (mitochondrial matrix targeted). Cells expressing the above-mentioned plasmids were imaged 24 h after transfection. GP was added to the cells for 16 h before imaging. Live-cell fluorescence ratiometric imaging using the respective Hyper and SypHer plasmids was done on Leica SP8 confocal microscope, at 37 °C and 5% CO_2_, using a 93 × glycerol objective (NA = 1.3) at 2 × zoom to acquire a field of view of 62.5 × 62.5 µm. Single optical plane images were obtained in a line sequential mode by excitation at 488 and 405 nm and the emission channel was obtained using a HyD detector from 495 to 545 nm. A line accumulation of 4 was used to obtain the images. 100 µM H_2_O_2_ was added to the A375 cells and cells were imaged within 30 min of H_2_O_2_ addition that served as a positive control for fluorescent H_2_O_2_ detection.

Fiji software was used to quantify the ratio images acquired at 488 and 405 nm, using a custom written macro, operating in a batch mode. Average pixel intensity for each cell was obtained by dividing the images obtained using 488 nm excitation by images obtained using 405 nm excitation after marking the boundary of the corresponding cell. A threshold was manually applied to the ratio images to exclude background pixels with zero intensity and calculation of average intensity was limited to pixels which were not excluded after the thresholding.

### Intracellular ROS (DCF assay)

Generation of intracellular reactive oxygen species (ROS) was measured using 2′,7′-dichlorodihydrofluorescein diacetate (H_2_DCF-DA). The cell permeable substance is cleaved by intracellular by esterases to H_2_DCF, which is further oxidized by ROS to the fluorescent DCF. The assay was performed in 24-well plates. Cells were incubated with 100 µM H_2_DCF-DA in Hanks’ Balances Salt Solution (HBSS) for 0.5 h, washed 2 × with HBSS and treated with different concentrations of GP or 2 mM H_2_O_2_ (positive control), in 0.5 ml HBSS. HBSS medium was used because components of DMEM were described to catalyze the production of H_2_O_2_, thus may interfering with the assay (Boulton et al. 2011; Brubacher and Bols 2001; Tetz et al. 2013). DCF fluorescence was measured with a plate reader in 5 min intervals (ex: 485 nm, em: 520 nm; FLUOstar OPTIMA, Germany). To calculate the formation of ROS, the basal ROS level was subtracted from the amount of ROS detected after 90 min. Mock-treated control was set at 1.

### Caspase activity assay

Caspase 9 activity or Caspase 3/7 activity were measured using Cell Meter™ Caspase 9 and Caspase 3/7 Activity Apoptosis Assay Kit (AAT Bioquest, Sunnyvale, California, USA), respectively. Cells were seeded in 96-well plates overnight and were incubated with different concentration of GP for different times. The alkaloid staurosporine (sts, 20 µM) was used as positive control and 80 µM Z-VAD(OMe)-FMK to inhibit caspase activity. The assay was performed according to the manufacture’s specifications. Mock-treated control was set at 1.

### SDS-PAGE and Western Blotting

For SDS (sodium dodecyl sulfate polyacrylamide gel) electrophoresis and Western Blotting (Laemmli 1970), cells were lysed after incubation with GP in 1% SDS (Roth, Karlsruhe, Germany) with 1:1000 protease inhibitor cocktail and sonicated. Protein concentration was determined with the DC™ Protein Assay Kit. 20 µg protein of each sample was mixed with 4 × SDS-PAGE sample buffer (40% glycerol, 20% β-mercaptoethanol, 12% SDS, 0.4% bromophenol blue) and heating at 95 °C for 10 min. Subsequently, the samples were subjected to 12% or 15% (w/v), respectively, SDS-polyacrylamide gels. After blotting of proteins onto polyvinylidene difluoride (PVDF) membranes (GE Healthcare, Solingen, Germany), the blot was developed using the ECL-system (Cell Signaling Technology, Frankfurt a. Main, Germany) and monitored by the Fusion SL Advance gel documentation device (Peqlab, Erlangen, Germany). Quantification of proteins was done by FusionCapt Advance software.

### Mitochondria isolation

Melanoma cells were incubated with GP for 6 and 24 h or with 20 µM staurosporine (sts) for 4 h as positive control. The subsequent fractionation is done with minor modifications as described earlier (Wieckowski et al. 2009). After incubation, cells were washed with cold PBS once and harvested with a cell scraper. The cells were centrifuged (500×*g*, 4 °C, 5 min), the supernatant was removed and cells were lysed with cold lysis buffer (210 mM mannitol, 70 mM sucrose, 1 mM EDTA, 20 mM HEPES, 1 × protease inhibitor) for 10 min on ice. The cell lysate was homogenized using Sterican® cannula (Ø 0.90 × 40 mm, 20 G) and centrifuged (600×*g*, 4 °C, 10 min). If the supernatant was turbid, the lysate was centrifuged again (1000×*g*, 4 °C, 10 min). The supernatant, containing the mitochondria, was transferred into a fresh tube and centrifuged (6500×*g*, 4 °C, 15 min). After that, the pellet (mitochondria fraction) was suspended in 50 µl lysis buffer. Protein concentration was determined as described above and equal amount of proteins (20 µg) were used for Western Blot analysis as mentioned above.

### RNA interference

For transient knockdown, subconfluent A375 melanoma cells were transfected with Drp1 siRNA (ID 19561, AM51331, Ambion, Thermo Fisher Scientific) or control siRNA (4404021, Ambion, Thermo Fisher Scientific) by a standard Lipofectamine RNAiMAX (Invitrogen) transfection procedure according to the manufacturer`s instruction for 48 h. After transfection, cells were lysed for Western Blotting for validation of the knockdown or mock-treated and treated with 2.5 µM GP, respectively, to determine the fragmentation of mitochondria as described previously.

### Statistics

Means were calculated from at least three independent experiments, unless otherwise stated. Error bars represent the standard error of mean (SEM). Statistical analysis was performed by one-way ANOVA with post hoc test or student’s *t* test; *p ≤ 0.05; **p ≤ 0.01 and ***p ≤ 0.001 as levels of significance.

## Results

### GP selectively decreases the viability of A375 melanoma cells

**Fig. 2:**
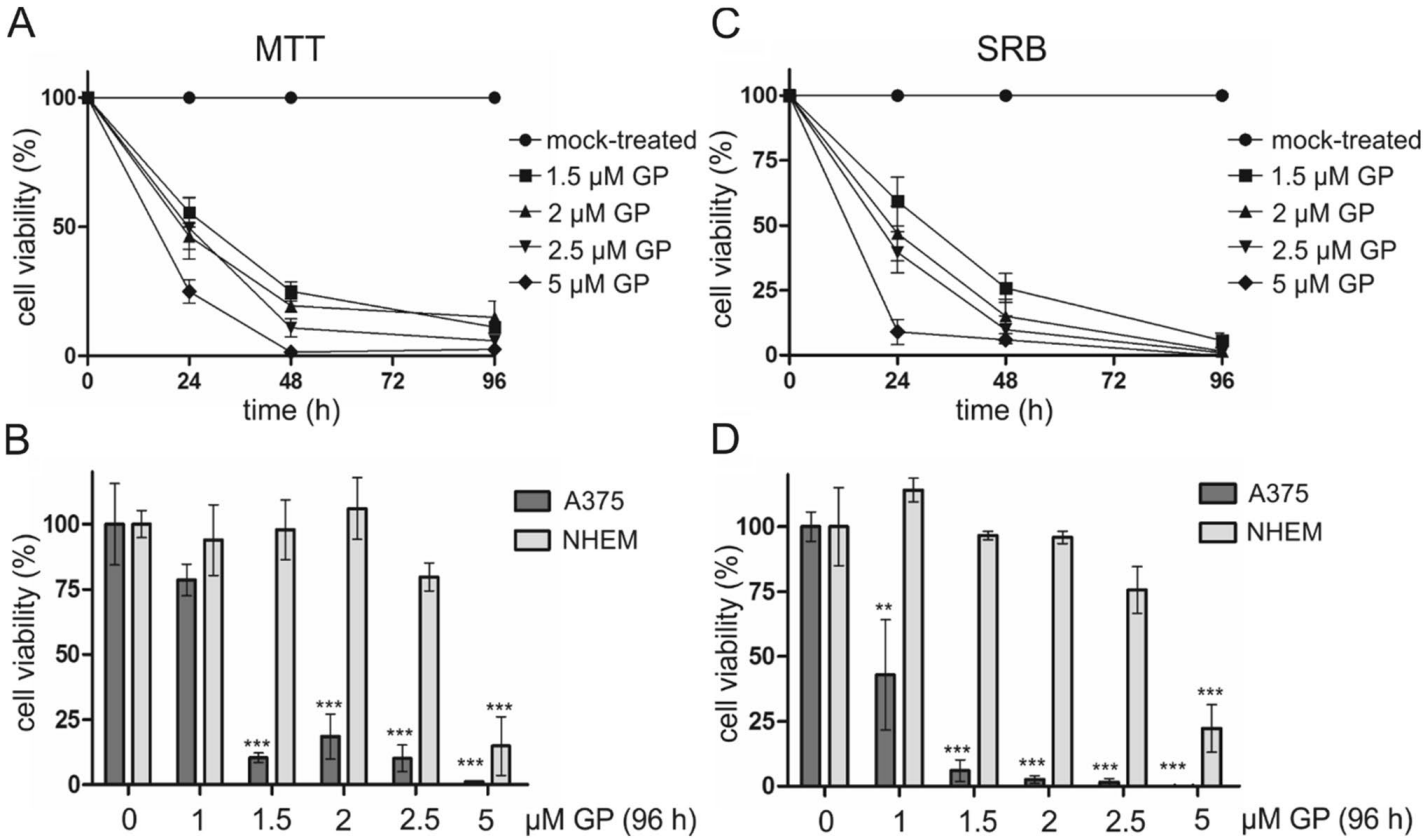
Effect of GP on the cell viability of A375 melanoma cells and NHEM melanocytes. **a**, **c** To determine the effect of GP on cell viability, A375 melanoma cells were treated with different concentrations of GP for 24, 48 and 96 h. Cell viability was determined with MTT (**a**, **b**) and SRB (**c**, **d**) assay. OD value of mock-treated control was set at 100%. **b**, **d** For comparison of A375 melanoma cells and NHEM melanocytes, cells were treated with different concentration of GP for 96 h. Data represent means ± SEM, *n* = 3–6. One-way ANOVA with Dunnett’s multiple comparison test was used for the determination of statistical significance. **p < 0.01, ***p < 0.001

The pan Bcl inhibitor (±) gossypol (GP) was tested in skin melanoma cells and healthy epidermal melanocytes (NHEM). GP lowered the cell viability of A375 melanoma cells in a concentration- and time-dependent manner as measured in the MTT assay. With the lowest concentration of 1.5 µM, a 40% decrease of cell viability was found after 24 h compared to mock-treated cells which were set at 100%. The strongest effect was observed in cells treated with 5 µM GP for 96 h, leading to a complete loss of viability (Fig. 2a). To test for the selectivity of GP, the effect of GP on NHEM melanocytes was also investigated (Fig. 2b, light grey bars). In contrast to tumor cells, GP showed no significant toxic effect up to a concentration of 2.5 µM GP at 96 h post-treatment in melanocytes. Only with the highest dose, a significant cell toxicity was observed in NHEM (Fig. 2b). Since the MTT assay provides information on metabolic activity and MTT itself is toxic to cells over time resulting in false-positive results, we used the SRB assay to verify the effect of GP on the cells (Riss et al. 2004). Here, GP significantly lowered the viability of A375 melanoma cells with increasing concentrations and over time (Fig. 2c). Again, the cytotoxic effect was significantly stronger in the tumor cells than in the melanocytes (Fig. 2d). Thus, the results from both assays and the IC_50_ values of GP after 96 h (Supplemental Fig. 1b, c), indicate that GP exerts a selective cytotoxicity on A375 melanoma cells. The IC_50_ concentration of GP in A375 melanoma determined with nonlinear regression analysis is 1.8 µM (SRB) and 2.1 µM (MTT) at 24 h (Supplemental Fig. 1a). A concentration of 2.5 µM GP was used for the majority of the following experiments because no significant toxicity on NHEM was observed at this level (Fig. 2b, d).

### Cellular uptake of GP

**Fig. 3:**
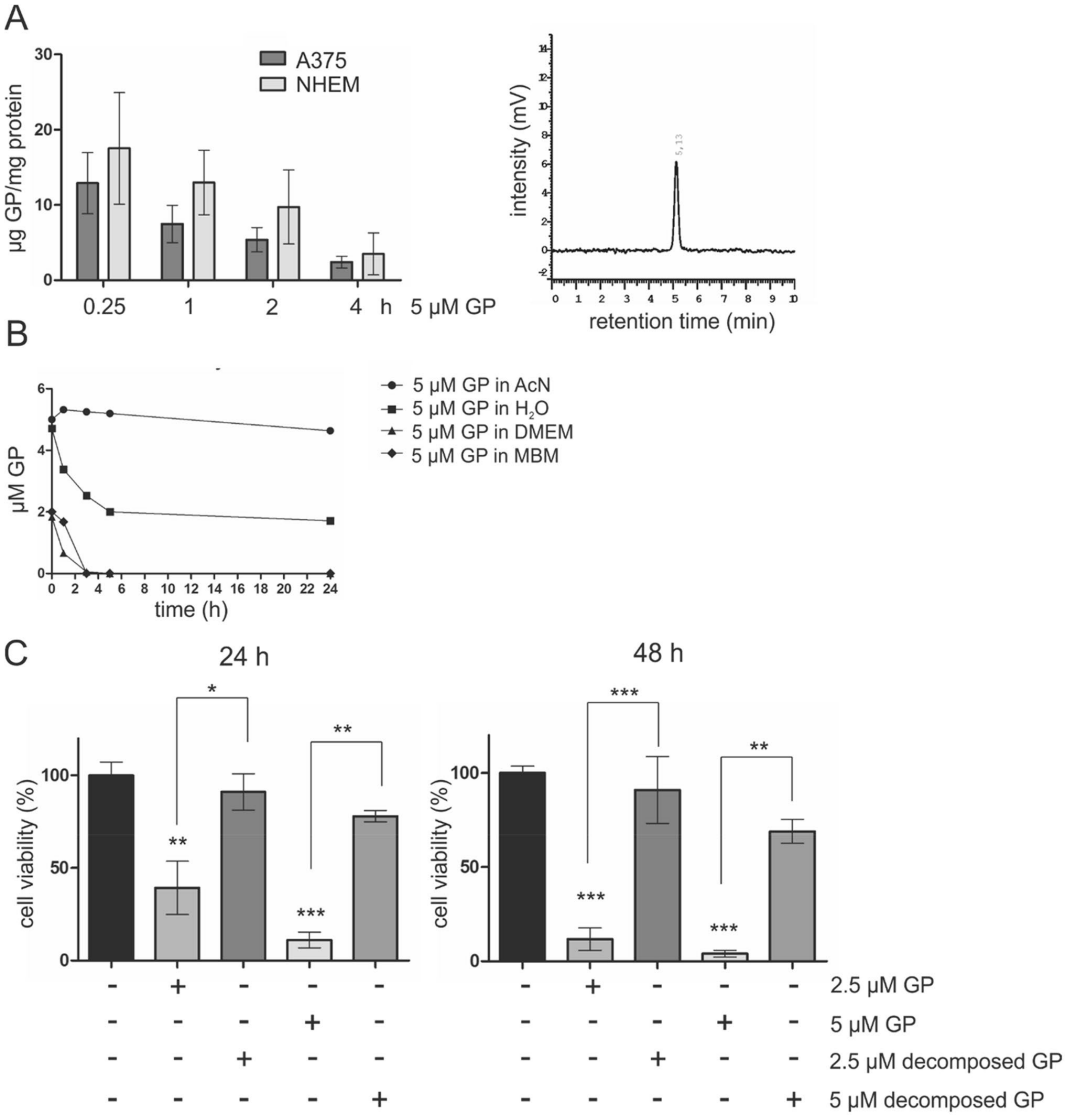
Cellular uptake, stability and effect of parent GP on cell viability. **a** Left panel: detection of intracellular GP content in A375 melanoma and NHEM melanocytes. After incubation with 5 µM GP for 0.25, 1, 2 and 4 h, cells were harvested and analyzed by HPLC. For quantification, the amount of intracellular GP was set in relation to the protein level. Data represent means ± SEM, *n* = 3. Right panel: a representative chromatogram of HPLC analysis. **b** Stability of GP was tested in different solutions (AcN, H_2_O, DMEM, melanocyte basal medium (MBM)) and were analyzed by HPLC at 367 nm. **c** Comparative study of the effect of parent and decomposed GP on cell viability in A375 melanoma using MTT assay. Cells were treated with 2.5 µM GP and 5 µM GP for 24 and 48 h directly or after decomposition of GP in DMEM, respectively. Cell viability of mock-treated cells was set at 100%. Data represent means SEM, *n* = 3. One-way ANOVA with Bonferroni’s multiple comparison test was used for the determination of statistical significance. * < 0.05, **p < 0.01, ***p < 0.001

To study if selective effect of GP is due to different intracellular concentration of GP in tumor versus normal cells, the cellular uptake was measured by HPLC. Within 15 min, GP was detected in the tumor cells as well as in melanocytes. The quantification of GP showed that the amount in the cell types did not differ substantially, but in both A375 and NHEM, the levels decreased within 4 h (Fig. 3a). We calculated the detectable amount of GP (*t* = 15 min) in the cells to the total amount added and were able to detect an average of around 15% of the amount applied. Interestingly, the amount of GP was consistently higher in the melanocytes at each concentration of GP.

### Parent GP is responsible for the cytotoxic effect

The stability of GP in different solvents was tested. While GP is stable in AcN over 24 h, almost 50% of the substance is decomposed in water and parent GP is completely lost after 5 h incubation in DMEM and melanocyte basal medium (MBM), respectively (Fig. 3b). The instability of GP in aqueous solutions raises the question whether the substance itself (parent compound) or its decomposition products (metabolites) mediate the cytotoxic effect on tumor cells. Therefore, GP was preincubated in medium until it was completely decomposed (5 h) and no longer detectable by HPLC. Subsequently, A375 melanoma cells were treated either with decomposed or parent GP for 24 and 48 h. In contrast to directly added GP, preincubated GP (decomposed GP, GP metabolites) did not significantly decrease the cell viability (Fig. 3c), which led to the conclusion that intact GP is responsible for the toxic effect in tumor cells.

### GP decreases mitochondrial membrane potential (Δ*Ψ*_*m*_) and induces fragmentation in A375 melanoma cells

**Fig. 4:**
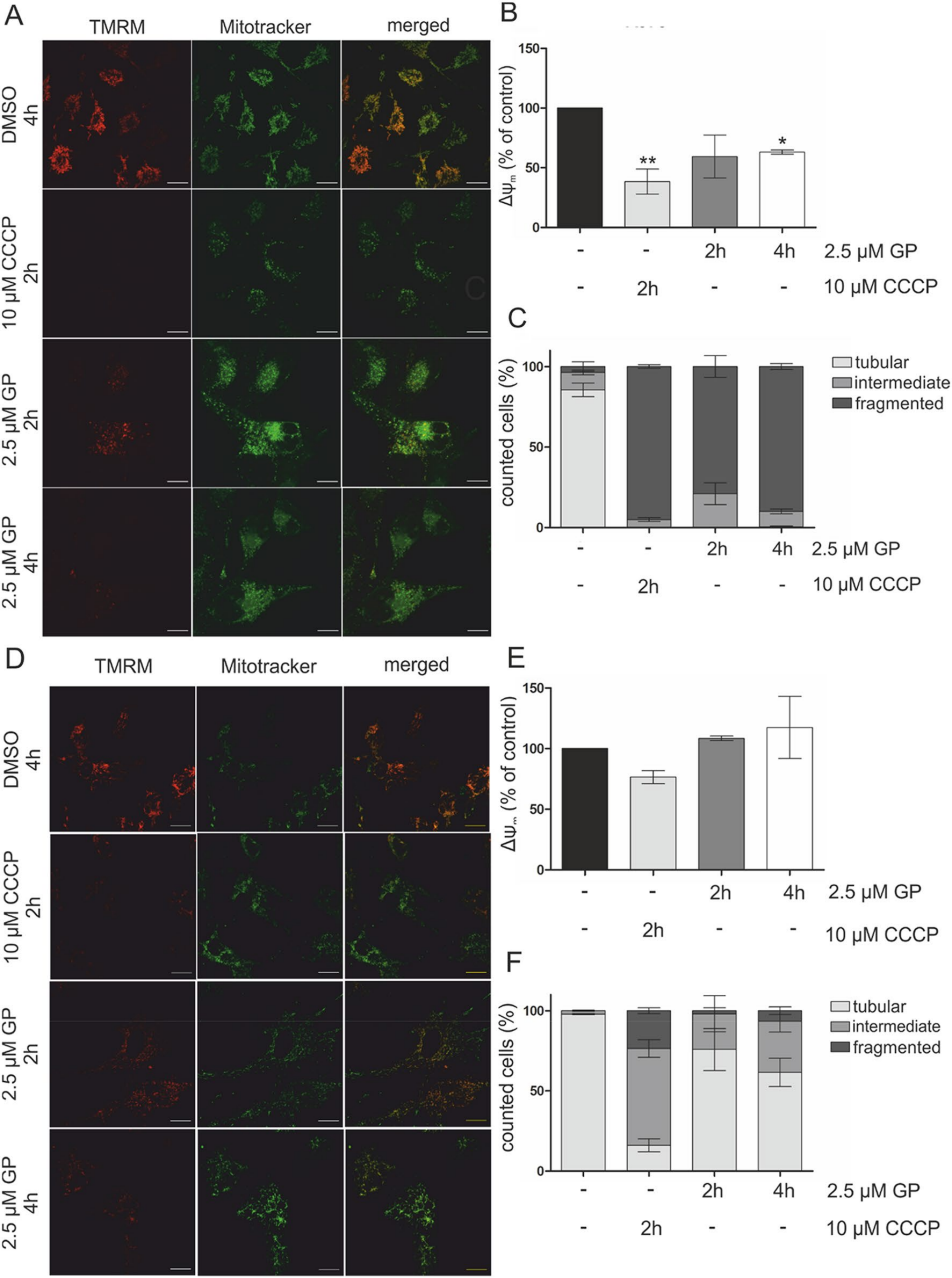
Decrease of mitochondrial membrane potential and morphological changes in mitochondria of A375 melanoma cells. Melanoma cells (**a**–**c**) and melanocytes (**d**–**f**) were mock-treated or treated with 2.5 µM GP for 2 and 4 h, respectively, or with 10 µM CCCP for 2 h as positive control. After incubation, 100 nM TMRM and 100 nM MitoTRACKER™ Green were added to the cells. **a**, **d** Representative pictures of three independent experiments are shown. **b**, **e** The quotients of the fluorescence intensity of TMRM and MitoTRACKER™ Green were calculated using Image J and the mock-treated control was set at 100%. One-way ANOVA with Dunnett’s Multiple Comparison Test was used for the determination of statistical significance. *p < 0.05, **p < 0.01. **c**, **f** At least 30 cells were used to quantify the mitochondrial morphology. Data represent means ± SEM, *N* = 3

BH3 mimetics are described to bind anti-apoptotic proteins, thus, shifting the balance of pro- and anti-apoptotic proteins and pro-apoptotic proteins (Lessene et al. 2008; Opydo-Chanek et al. 2017). In A375 cells, treatment with 2.5 µM GP for 24 h led to a change in the ratio of mitochondrial and cytosolic BAX towards the mitochondria compartment as shown in the supplementary Fig. 2. The ratio of mitochondrial to cytosolic BAX is increased after 24-h treatment of GP in favor of the mitochondrial fraction. This confirms the postulated mechanism of GP as a BH3 mimetic. To assess whether GP has an effect on mitochondrial integrity, the mitochondrial membrane potential (*ΔΨ*_*m*_) in A375 melanoma cells and NHEM melanocytes was analyzed using confocal microscopy. Even after 4 h of GP incubation, the *ΔΨ*_*m*_ was significantly lowered in A375 melanoma cells as assessed by weakening of the signal of the mitochondrial membrane sensitive TMRM staining. The uncoupler CCCP used as positive control likewise showed a decrease of the fluorescent dye TMRM. MitoTRACKER™ green was used as dye for mitochondria staining (Fig. 4a). Figure 4b shows the image analysis where TMRM staining was normalized to MitoTRACKER™ and the control was set at 100%. As shown earlier *ΔΨ*_*m*_ is affected by mitochondrial dynamics (Legros et al. 2002; Tang et al. 2018), and the effect of GP on mitochondrial dynamics was investigated by analyzing the fragmentation of mitochondria. GP induced an almost complete fragmentation of mitochondria in A375 melanoma cells. Nearly, 80% of mitochondria were fragmented after 2 h and about 90% after 4 h, similar to the positive control CCCP (Fig. 4c). No tubular structures were apparent in the presence of GP. In contrast, GP did not change the *ΔΨ*_*m*_ in normal melanocytes (Fig. 4d, e) and had only a modest increase in percentage of cells possessing mitochondria having intermediate morphology (Fig. 4f).

### Mitochondrial fragmentation is dependent on Drp1 expression

**Fig. 5:**
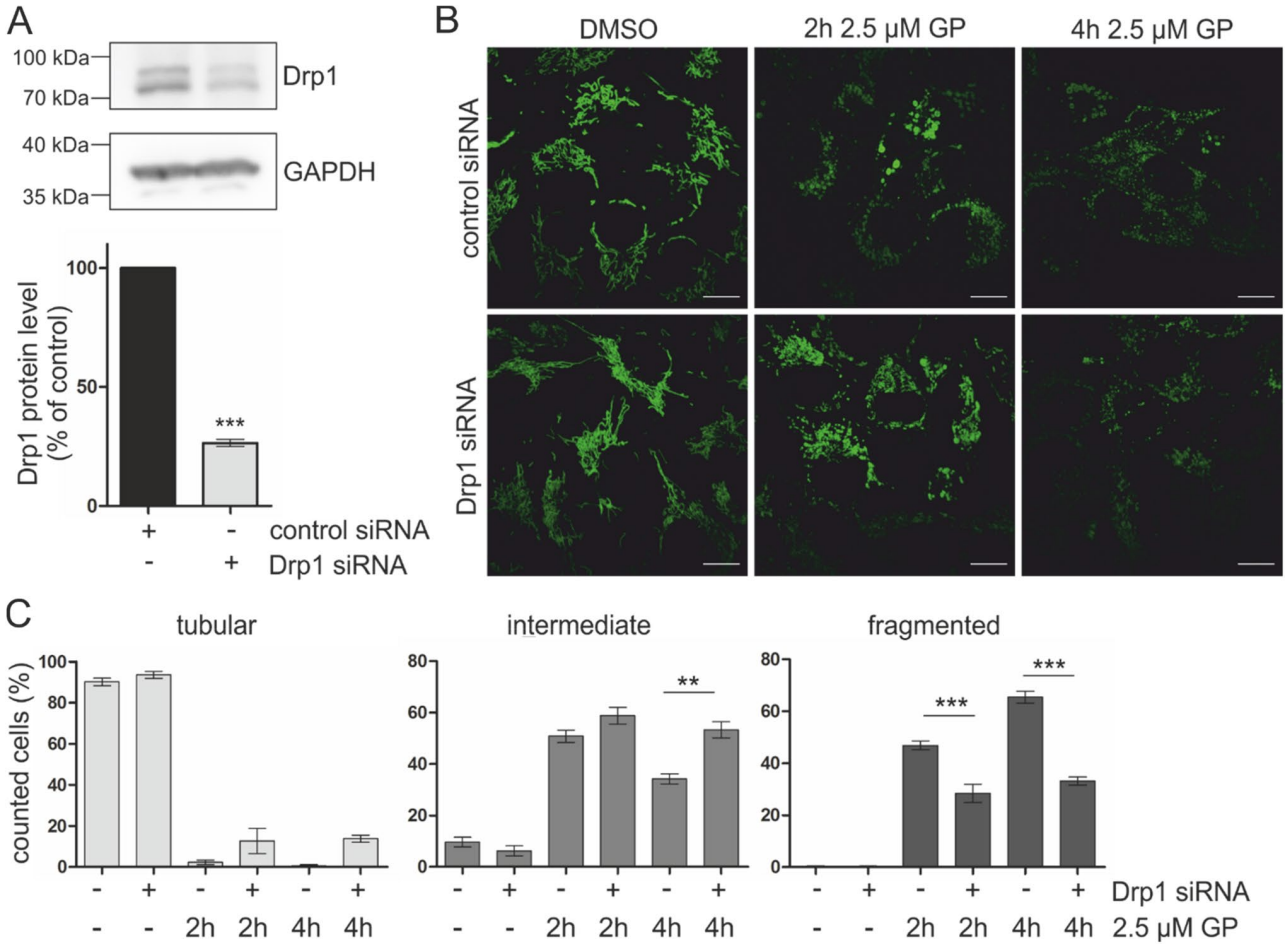
Drp1 knockdown in A375 melanoma cells. **a** Subconfluent A375 melanoma cells were knocked down for Drp1 and used for SDS-PAGE and Western Blotting for validation of knockdown efficiency. A representative Blot of three independent experiments is shown. Densitometry was calculated using FusionCapt advance software. The protein expression of control was set at 100%. GAPDH was used as loading control. Data are means ± SEM, *n* = 3. Student’s *t* test was used for the determination of statistical significance. ***p < 0.001. **b** After Drp1 knockdown, cells were mock-treated or treated with 2.5 µM GP for 2 or 4 h. The mitochondrial morphology was assessed by fluorescence microscopy. Representative pictures are shown. **c** Quantification of mitochondrial morphology in A375 melanoma cells (tubular, intermediate, fragmented). Data represent means ± SEM, *N* = 3, *n* < 50. One-way ANOVA with Bonferroni’s multiple comparison test was used for the determination of statistical significance. **p < 0.01, ***p < 0.001

Due to the strong effect of GP on the mitochondrial network in A375 cells, we investigated whether Drp1, a GTPase acting as key regulator of mitochondrial fission (Tilokani et al. 2018), is involved in this process. Hence, A375 melanoma cells were knocked down for Drp1. Knockdown efficiency (75%) is shown in Fig. 5a. After transfection, 2.5 µM GP was added to the cells and mitochondria morphology was examined. Despite the Drp1 knockdown, the mitochondrial morphology is still affected by after GP treatment. However, the percentage of cells having intermediate state increased significantly after 4 h with a concomitant decrease of fragmented cells at both time points (Fig. 5b, c). This indicates that the GP-initiated mitochondrial fragmentation is at least partly dependent on Drp1.

### ROS are not involved in GP-mediated effects

**Fig. 6:**
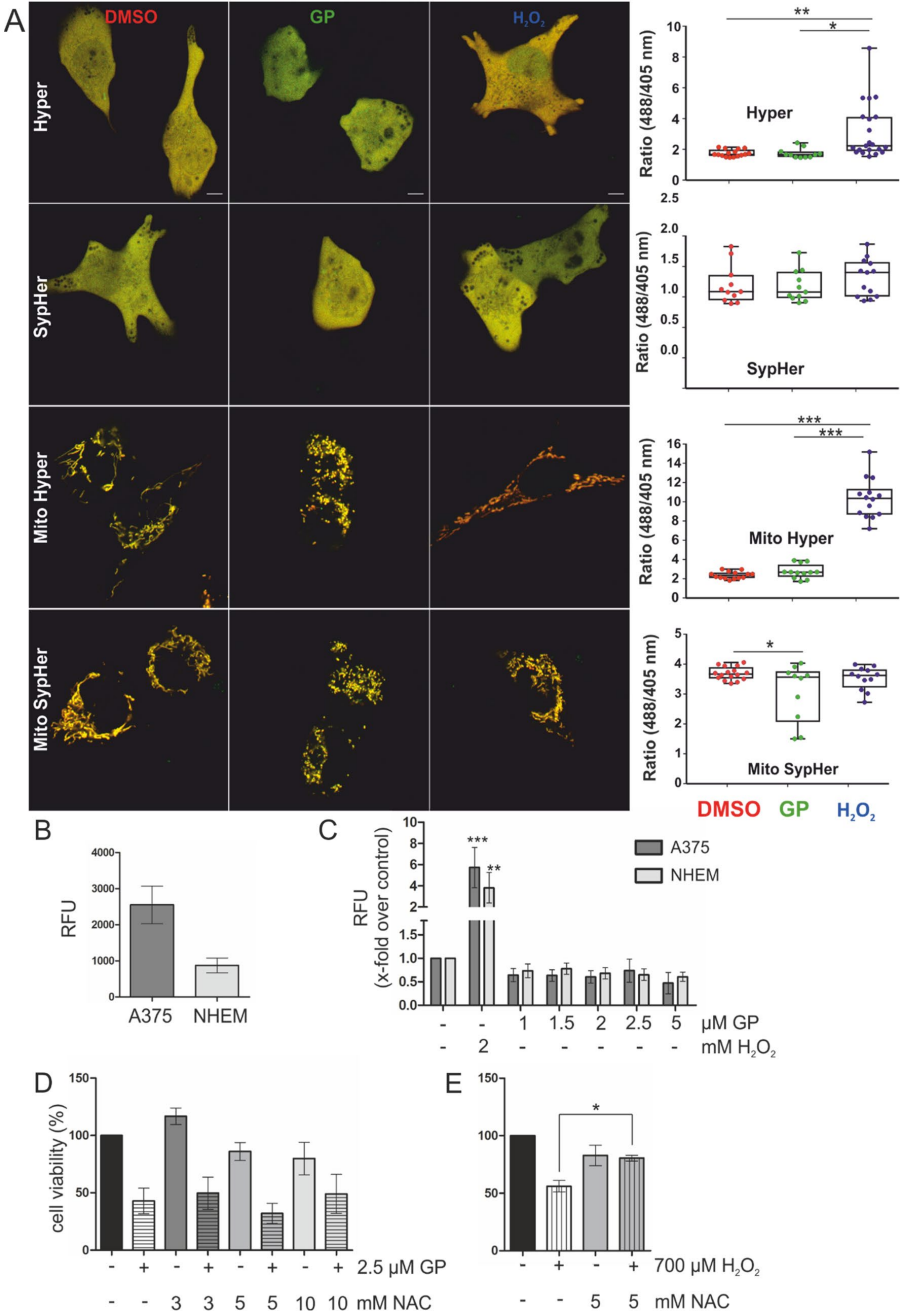
Effect of GP on generation of ROS in A375 melanoma and NHEM melanocytes. **a** Left panel: subconfluent A375 melanoma cells were transiently transfected with one of the four plasmids: Hyper (cytosolic), SypHer (cytosolic), mito-Hyper (mitochondrial matrix targeted) and mito-SypHer (mitochondrial matrix targeted). Cells were mock-treated or treated with 2.5 µM GP for 16 h or 100 µM H_2_O_2_. Corresponding merged images of both channels are shown. Right panel: box plot shows the quantification of H_2_O_2_ levels of each cell in different conditions. Each point represents the average intensity of ratiometric fluorescence of each cell. Student’s *t* test was used for comparison of different conditions. **b**, **c** To determine the intracellular ROS generation, the DCF assay was performed. After incubation with 100 µM H_2_DCF-DA for 30 min, A375 melanoma and NHEM melanocytes were treated with different concentration of GP (1–5 µM) in HBSS and the fluorescence was immediately measured in 5-min intervals for 90 min. **b** Basal ROS levels of A375 and NHEM were presented as relative fluorescence units (RFU) of the first measuring point. **c** The amount of generated ROS was calculated by subtracting the RFU of the starting point from the last measuring point. The mock-treated control of each cell type was set at 1. H_2_O_2_ (2 mM) served as positive control. **d**, **e** To confirm a ROS-independent mechanism of GP, A375 melanoma were pretreated with 3, 5 and 10 mM of the antioxidant n-acetyl-L-cysteine (NAC) for 4 h. Thereafter, 2.5 µM GP (**d**) and 700 µM H_2_O_2_ (positive control, e), respectively, was added for further 24 h. The cell viability was measured by MTT assay and the mock-treated control was set at 100%. Student’s *t* test was used for the determination of statistical significance between H_2_O_2_ and H_2_O_2_ + NAC treatment *p < 0.05. Data represent means SEM, *n* = 3–5

Loss of *ΔΨ*_*m*_ and mitochondrial fission are linked with formation of ROS (Korshunov et al. 1997; Serasinghe and Chipuk 2017; Yu et al. 2006). We investigated if GP-induced fragmentation is accompanied with ROS formation. In this context, we used Hyper, a genetically encoded fluorescence-based ratiometric probe for detecting the levels of H_2_O_2_ (Bilan and Belousov 2016; Booth et al. 2016b). Hyper is a redox sensor containing a bacterial H_2_O_2_-sensing domain (OxyR) and a circularly permuted Yellow Fluorescence Protein (cpYFP). Oxidation of cysteine 199 by H_2_O_2_ changes the confirmation of the flexible OxyR domain rendering a change in the fluorescence emission spectrum of cpYFP. Hence, there is a decrease and increase of fluorescence at shorter and longer wavelength emission, respectively, in the presence of H_2_O_2_. To account for H_2_O_2_ changes caused by alterations in pH, SypHer is routinely used as demonstrated earlier (Booth et al. 2016a). SypHer has a single mutation in C199S compared to Hyper and serves as a redox insensitive probe. We used Hyper and SypHer (present in the cytosol) and mitochondrial matrix-targeted versions of Hyper and SypHer (mito-Hyper and mito-SypHer) to check the subcellular redox status. Hence, genetically encoded fluorescent redox indicators have the advantage of being targeted to specific intracellular compartments (Weller et al. 2014). When A375 melanoma cells expressing Hyper, SypHer, mito-Hyper and mito-SypHer were either mock-treated or treated with 2.5 µM GP for 16 h, we found no significant increase of H_2_O_2_ in the mitochondria and the cytosol (Fig. 6a) compared to control, indicating that the loss of MMP and the fragmentation is independent of ROS formation. Addition of 100 µM H_2_O_2_ led to a significant increase of ratiometric fluorescence validating our experiment (Fig. 6a).

To confirm a ROS-independent mechanism of GP, we focused on the formation of intracellular ROS. To determine the intracellular ROS level in GP-treated A375 melanoma and NHEM melanocytes, the H_2_DCF-DA assay was performed. Our data showed a higher basal ROS level in A375 melanoma cells than in NHEM melanocytes (Fig. 6b). However, no additional ROS have been generated after 90 min of GP treatment in both cell types. As positive control, the cells were treated with H_2_O_2_ (Fig. 6c). In a further experimental approach, A375 cells were preincubated with different concentrations of the antioxidant *N*-acetyl-l-cysteine (NAC). The treatment did not protect the tumor cells from the GP-dependent decrease of cell viability (Fig. 6d) suggesting that ROS do not play an essential role in the observed GP effects. As proof that the chosen concentration of NAC is suitable to cause a protective effect against H_2_O_2_-induced toxic effects, A375 cells were preincubated with 5 mM NAC and subsequently treated with 700 µM H_2_O_2_ for further 24 h (Fig. 6e). That finally resulted in a significant rescue of cell viability. To substantiate those findings, the antioxidant trolox, a water-soluble derivate of vitamin E, was tested as well. In accordance with the results of NAC preincubation, we did not observe a protective effect of trolox respect to GP treatment, although we have shown that the chosen concentration is sufficient to rescue the toxic effect of H_2_O_2_ on cell viability (Supplemental Fig. 3).

### Smac/DIABLO release after GP treatment

Although no ROS are formed after GP treatment, both a drop in mitochondrial membrane potential and mitochondrial fission was found to be linked to an apoptotic cell death (Ly et al. 2003; Suen et al. 2008). Especially the release of inner mitochondrial proteins such as Smac/DIABLO indirectly induces apoptosis through the inactivation of inhibitory apoptotic proteins (IAP), thus promoting the activation of caspases (Chai et al. 2000; Du et al. 2000; Verhagen et al. 2000). As the cytotoxic effect of GP has only been observed in the melanoma cells, the following studies were only performed with these. We assessed whether GP causes a release of Smac/DIABLO in A375 melanoma cells by analyzing the subcellular localization of this protein. GP caused a release of Smac/DIABLO from mitochondria into the cytosol with a significantly fivefold increase of the protein after 6 h post-treatment and a tenfold increase after 24 h compared to mock-treated cells, as determined by densitometric analysis of the Western Blot (Supplemental Fig. 4).

### GP triggers caspase-dependent apoptosis in A375 melanoma cells

**Fig. 7:**
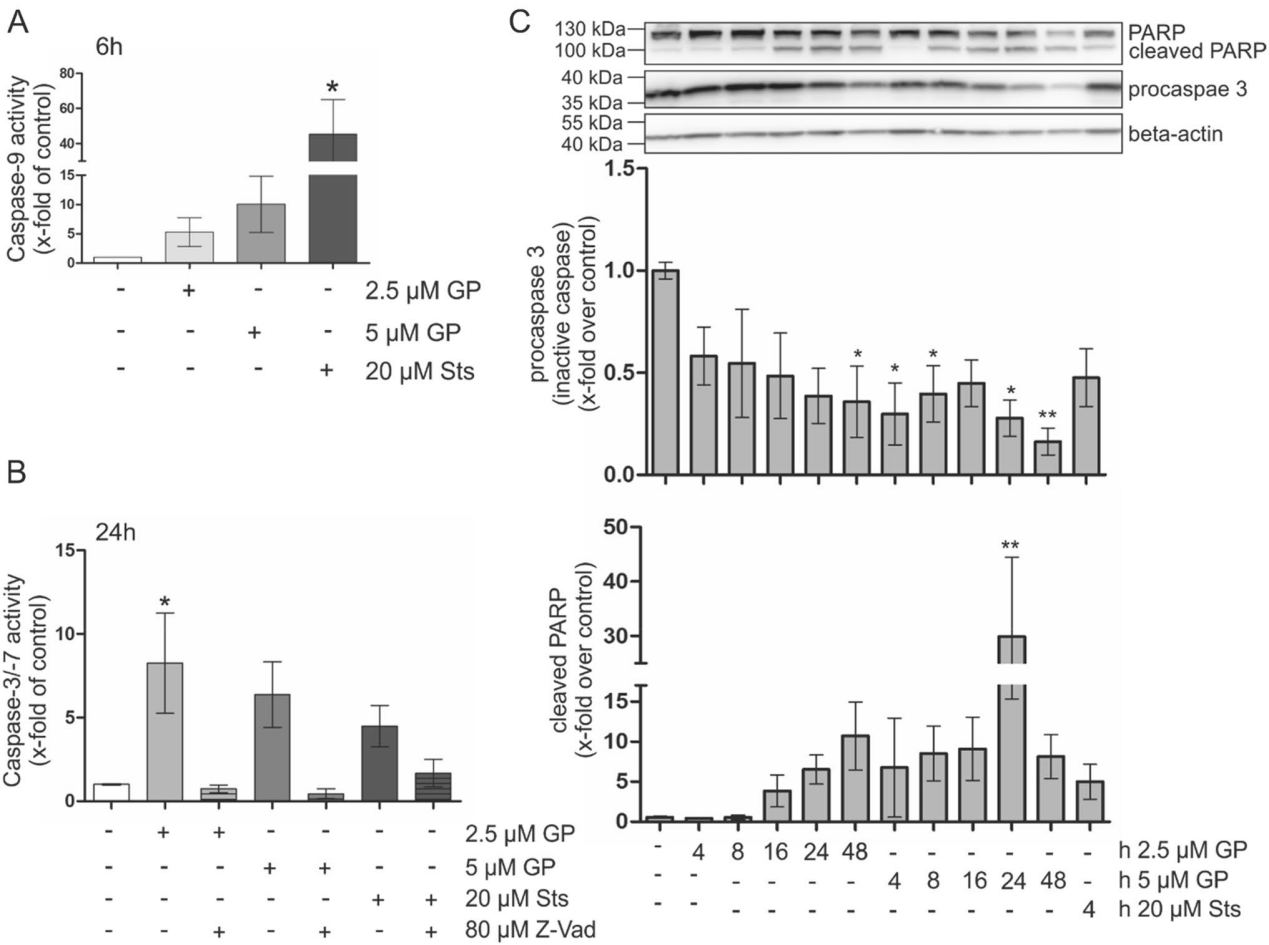
GP-dependent induction of caspase-dependent apoptosis in A375 melanoma via GP treatment. **A** The activity of the initiator caspase 9 was measured after 2.5 µM or 5 µM GP for 6 h. Staurosporine (sts, 20 µM) served as positive control in all experiments. Mock-treated control was set at 1. **b** After incubation of 2.5 µM and 5 µM GP, respectively, or mock treatment for 24 h, effector caspase 3/7 activity was measured. To prevent caspase activity, the pan caspase inhibitor Z-Vad was added 10 min before the end of the incubation time. **c** Western Blotting analysis of PARP cleavage and loss of procaspase 3. After incubation with 2.5 µM and 5 µM GP for different time points, cells and the supernatant were lysed and used for SDS-PAGE and Western Blotting. Quantification of cleaved PARP and procaspase 3 was determined by Image J. The x-fold increase of protein level compared to mock-treated control is presented. One-way ANOVA with Dunnett’s comparison test was used for the determination of statistical significance. *p < 0.05, **p < 0.01

As Smac/DIABLO release, an early apoptotic marker, was detected, late apoptotic markers including caspase activity, loss of (the inactive) procaspase 3, as well as PARP cleavage were analyzed in A375 melanoma cells. Treatment with GP at a concentration of 2.5 µM resulted in a fivefold increase of the activity of the initiator caspase 9 over the control level after 6 h and 5 µM GP led to an about tenfold activity increase (Fig. 7a). After 24 h, GP treatment was followed by an at least fivefold increase in caspase 3/7 activity at concentrations of 2.5 and 5 µM which was completely prevented by adding 80 µM of the pan caspase inhibitor Z-Vad (Fig. 7b). In addition, about 50% loss of procaspase 3, the inactive form of caspase 3, was observed in a time-dependent manner after GP treatment and the amount of cleaved PARP increased accordingly in a concentration- and time-dependent manner (Fig. 7c).

**Fig. 8:**
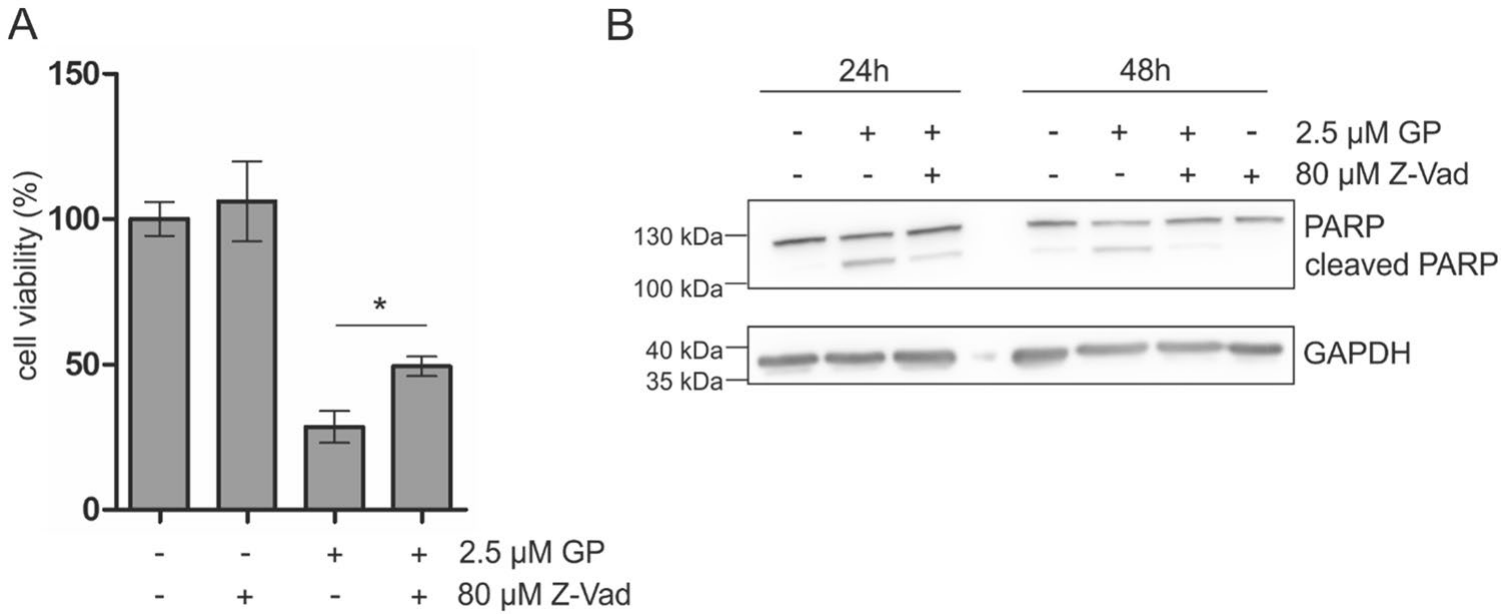
Rescue experiment with Z-Vad after GP treatment. After preincubation of 80 µM Z-Vad for 4 h, A375 cells were mock-treated or treated with 2.5 µM for further 24 h. **a** Cell viability was measured by the MTT assay. The mock-treated control was set at 100% viability. Student’s *t* test was used for the determination of statistical significance between GP and GP + Z-Vad treatment. **p < 0.01. Data are means ± SEM, *n* = 3. **b** Western Blotting analysis of PARP cleavage after GP treatment alone or in combination with Z-Vad. GAPDH served as positive control. A representative blot was depicted; *n* = 3

To confirm that GP-initiated apoptosis is responsible for GP-mediated cytotoxicity, a “rescue” experiment was performed. The cell viability of A375 melanoma was analyzed after preincubation with the pan caspase inhibitor Z-Vad using MTT assay (Fig. 8a). The coincubation resulted in a significant partial rescue of GP-mediated lowering of cell viability indicating a pivotal role of GP-dependent apoptotic cell death of A375 melanoma cells. In addition to that, the coincubation of Z-Vad and GP prevented PARP cleavage (Fig. 8b).

## Discussion

A major challenge in current cancer therapy is still the effective elimination of malignant melanoma as the present therapeutically approaches often have an only moderate response rate caused by resistance formation, recurrence and severe side effects (Chapman et al. 2011; Flaherty et al. 2012). Fostering the intrinsic way of apoptosis with BH3 mimetics is one approach to overcome resistant tumor cells as these agents antagonize the pro-survival Bcl-2 proteins, thus inducing cell death (Mohammad et al. 2015). It was reported that melanoma cells show higher basal expression of anti-apoptotic Bcl-2 proteins including MCL-1, Bcl-2 and Bcl-xL (Vogler 2014). (±) Gossypol (GP), a natural compound from cotton seed, acts as pan-Bcl-2 inhibitor leading to the induction of apoptotic cell death (Opydo-Chanek et al. 2017; Zeng et al. 2019). However, apart from the mode of action of possible anticancer drugs on the tumor cells, it is equally as important to gain insight on their effects on normal (healthy) cells concerning adverse effects of drugs on healthy tissue.

In the present study, we investigated the effect of different concentrations of GP on cytotoxicity and the underlying mechanism in melanoma cells as well as in normal (healthy) human epidermal melanocytes (NHEM). Focus was to determine concentrations of GP which promote selective cytotoxicity in tumor cells and avoid undesired cell death of normal (healthy) cells (here melanocytes). Here, we show that GP (at levels of 2.5 µM) lowers cell viability in a dose- and time-dependent manner in tumor cells, whereas normal cells are less vulnerable. These data are consistent with the literature which reported that healthy cells are more resistant to GP treatment. It has been shown that treatment with 5 µM (−) GP (AT-101) for 5 days did not affect the viability of oral keratinocytes (Wolter et al. 2006) and human fibroblasts cell lines (Oliver et al. 2004). In addition to that, the IC_50_ value of (±) GP treatment for 5 days was around 20 µM for breast epithelial cells (Jaroszewski et al. 1990).

In our study, the cellular uptake of GP into the cells did not differ between normal and malignant cells, suggesting that this is not the reason for the differences in cell viability between the two cell types. GP was not very stable in aqueous solutions raising the questions whether a decomposition or oxidation product of GP caused the effect on cell viability in A375 melanoma cells. Most studies dealing with the stability of GP only tested its stability in different solvents, but not in standard medium (Wang et al. 2019). For that reason, complete decomposed GP was added to the cells and cell viability was measured by MTT assay. Interestingly, the decomposed GP showed no effect on cell viability leading to the conclusion that the “parent” compound GP caused the cytotoxic effect. We showed for the first time that the exposure to intact GP is essential for its effectiveness on A375 melanoma.

Since the cellular uptake of GP is similar in both A375 melanoma cells and NHEM melanocytes, the question rises why GP has a selective toxic effect on tumor cells. One explanation could be oxidative stress, since tumor cells have an increased basal ROS content compared to healthy cells and are, therefore, more susceptible to further ROS formation via, for example, nanoparticles or H_2_O_2_ (Alili et al. 2013; Aplak et al. 2020). It has been reported that GP has a pro-oxidative effect in breast, pancreatic and prostate cancer (Zubair et al. 2016) as well as in multiple myeloma (Xu et al. 2014). Therefore, we measured the ROS level in A375 melanoma and NHEM melanocytes after 90 min GP treatment. Surprisingly, our data showed neither in the tumor cells nor the healthy cells an additional increase in the intracellular ROS level after GP treatment. In line with this finding, we did not detect H_2_O_2_ generation even after 16 h in A375 melanoma and the antioxidants NAC and, in that context, the antioxidant trolox did not protect the cells against the toxic effect of GP. Lack of GP-dependent ROS formation is probably due to the characteristics of the cells. Some studies have shown that exposure to GP did not increase ROS in some cancer types such as human leukemia cells and malignant mesothelioma (Benvenuto et al. 2018; Hou et al. 2004).To our knowledge, a ROS-independent mechanism of GP on melanoma cells has not been described before.

As ROS seem to be not involved in the cytotoxic effect of GP on melanoma cells, the role of GP on mitochondria was studied because members of the Bcl-2 family interacting with GP have been described to play a crucial role in the maintenance of mitochondrial integrity and regulation of the intrinsic apoptotic pathway (Henz et al. 2019; Pena-Blanco and Garcia-Saez 2018). BH3 mimetics bind to anti-apoptotic proteins of the Bcl-2 family, thereby allowing pore formation and release of pro-apoptotic proteins such as Smac/DIABLO (Geserick et al. 2014; Hu et al. 2012). Here, we found that GP significantly decreases the mitochondrial membrane potential (Δ*Ψ*_*m*_) already after 4 h in A375 melanoma cells, whereas no loss of Δ*Ψ*_*m*_ was observed in melanocytes. A drop in mitochondrial membrane potential was reported to be an early and necessary event in apoptosis, although it is controversially discussed whether changes in Δ*Ψ*_*m*_ are a prerequisite or only a consequence of the release of mitochondrial proteins into the cytosol (Deng et al. 2013; Heiskanen et al. 1999; Ly et al. 2003; Madesh et al. 2002). In addition to the lowered mitochondrial membrane potential, we observed a complete GP-dependent fragmentation of mitochondria. Several studies demonstrated a relationship between mitochondrial fission (fragmentation) and induction of apoptosis mediated by Drp1 and Fis1 (Autret and Martin 2009; Frank et al. 2001; Suen et al. 2008). Even though treatment with BH3 mimetic substances usually causes apoptosis and is accompanied by a mitochondrial response (e.g. release of proteins, changes in mitochondrial morphology, loss of *ΔΨ*_*m*_), only a few studies have investigated the role of Drp1 in mitochondria integrity. Milani and coworkers showed that several BH3 mimetics (ABT-199, A1331852, A1210477) induced mitochondrial fission and apoptosis in a Drp1-dependent manner (Milani et al. 2019, 2017). Appropriately, the treatment of human cardiomyocytes with the MCL-1 inhibitor S63845 resulted in Drp1-dependent mitochondrial fragmentation (Rasmussen et al. 2020). Conversely, Drp1 siRNA or the negative mutant Drp1 K38A did not affect the ability of the BI97CIqa and BI112D1, derivatives of GP, to induce mitochondrial fragmentation in the non-small cell lung cancer cell line H23 (Varadarajan et al. 2013). Furthermore, ABT-263 and A1210477 diminished the active Drp1 form in melanoma and the inhibition of Drp1 by M-Divi enhanced the cytotoxicity of these compounds (Mukherjee et al. 2018). Here, downregulation of Drp1 using siRNA significantly lowered the percentage of fragmented mitochondria suggesting that the effect is at least partly dependent on Drp1. In addition, the fragmentation is not associated with increased ROS formation as no H_2_O_2_ was detected in melanoma cells expressing genetically encoded fluorescent ratiometric sensor Hyper in both the cytosol and mitochondria.

However, GP treatment led to a shift of the pro-apoptotic BAX from the cytosol to the mitochondria. In general, BH3 mimetics were designed to induce apoptosis. Therefore, we explored further markers of apoptosis and showed that GP induced release of Smac/DIABLO from the mitochondria into the cytosol as well as the activity of the initiator caspase 9. Concomitantly, loss of procaspase 3 (inactive caspase 3), enhanced activity of caspase 3 and increased PARP cleavage were observed over time in our study. These results are consistent with the literature which reported that 10 µM (−) GP led to Smac/Diablo dependent apoptosis in chemoresistant ovarian cancer cells (Hu et al. 2012). In addition to that, Benvenuto and coworkers showed that 12.5 µM (−) GP induced cleavage of several caspases and PARP after 48 h in malignant mesothelioma (Benvenuto et al. 2018). To confirm that caspase-dependent apoptosis is responsible for the effect on cell viability, the A375 melanoma cells were preincubated with the caspase inhibitor Z-Vad before GP treatment. The partial rescue of cell viability as well as the diminished PARP cleavage by Z-Vad indicates a GP-initiated caspase-dependent cell death in A375 melanoma cells. Thus, our data suggest that the loss of mitochondrial membrane potential as well as the almost complete, and partial Drp1-dependent fragmentation of the mitochondria by GP treatment is accompanied by the intrinsic pathway of apoptosis supported by release of the mitochondrial protein Smac/DIABLO.

In conclusion, GP has a selective toxic effect on A375 melanoma cells while normal (healthy) melanocytes are less vulnerable to the noxious agent. The different susceptibility to the substance within a certain concentration range may offer the possibility of a therapeutic window for the treatment of melanoma in vivo without affecting healthy cells. We showed that the mechanism of cytotoxicity of GP underlies its initial structure, as its decay completely prevented the effect on cell viability in tumor cells. Our results imply that GP induces ROS-independent but mitochondria-dependent apoptosis and a concomitant mitochondrial dysfunction in A375 melanoma cells in vitro*.*

## Supplementary Information

Below is the link to the electronic supplementary material.

Supplementary file1 (DOCX 213 KB)

Supplementary file2 (DOCX 137 KB)

Supplementary file3 (DOCX 100 KB)

Supplementary file4 (DOCX 202 KB)

## Data Availability

The datasets generated during and/or analyzed during the current study are available from the corresponding authors on reasonable request.
