# Supplementary material for: The BH3 mimetic (±) gossypol induces ROS-independent apoptosis and mitochondrial dysfunction in human A375 melanoma cells in vitro
Source: Arch Toxicol. 2021 Feb 1;95(4):1349–65. doi: 10.1007/s00204-021-02987-4 (PMC8032633; doi:10.1007/s00204-021-02987-4)
Supplement: Supplementary file 1 — Supplementary file1 (DOCX 213 KB) [file 204_2021_2987_MOESM1_ESM.docx]

# **The BH3 mimetic** **(±) gossypol induces ROS-independent apoptosis and mitochondrial dysfunction in human A375 melanoma cells *in vitro***

Lisa Haasler*^1^, Arun Kumar Kondadi^1^, Thanos Tsigaras^1^, Claudia von Montfort^1^, Peter Graf^1^, Wilhelm Stahl^1^, Peter Brenneisen^1^

^1^Institute of Biochemistry and Molecular Biology I, Medical Faculty, Heinrich Heine University Düsseldorf, Düsseldorf, Germany

*Corresponding author, e-mail: lisa.scharf@hhu.de


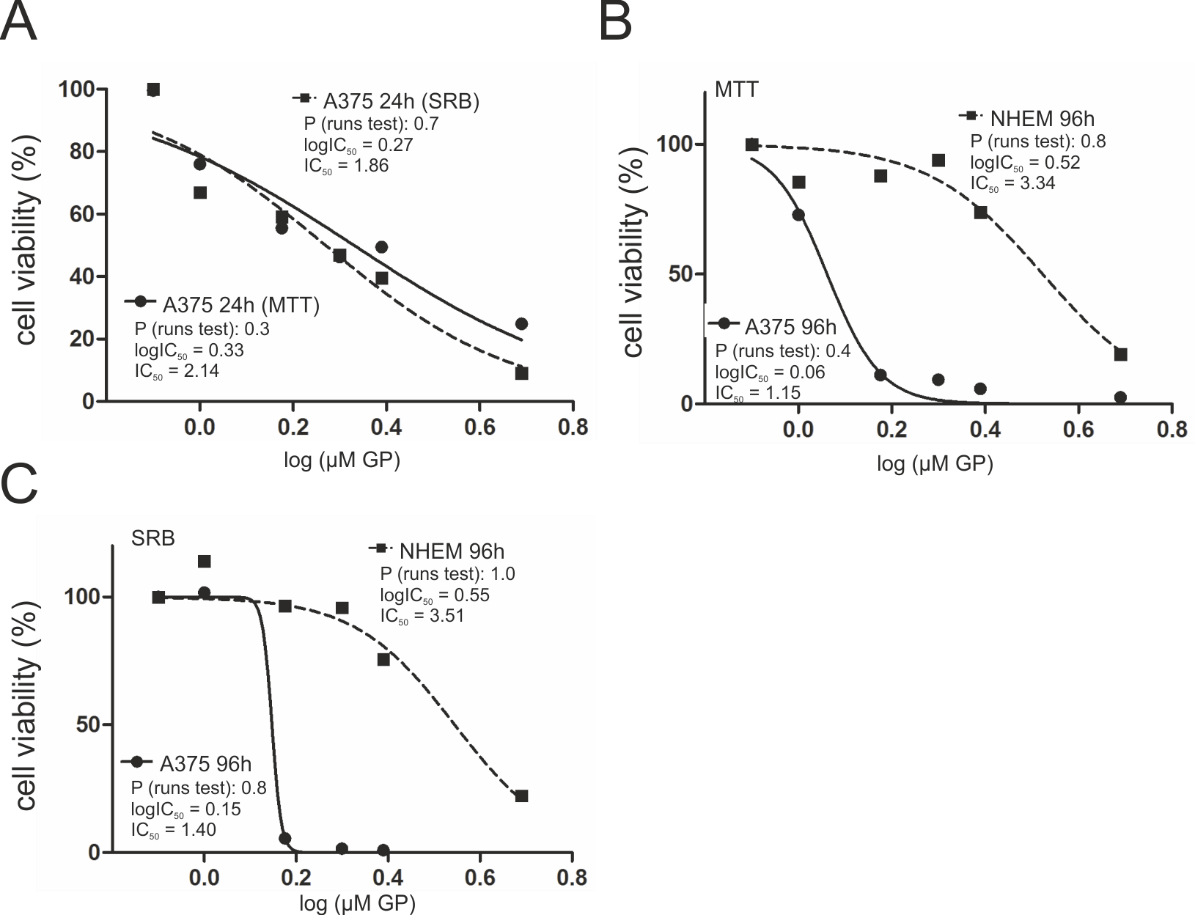


**Supplemental figure 1:** Calculation of IC50 value after GP treatment in melanoma cells (A375) and normal epidermal melanocytes (NHEM). A375 cells were treated with different concentration of GP for 24h using MTT (continuous line) and SRB assay (dotted line) (**a**). Cell viability on A375 and NHEM after GP treatment for 96h were determined with MTT (**b**) and SRB (**c**) assay. The means of at least three independent experiments (shown in Fig.2) were depicted. IC50 value of melanoma and melanocytes were calculated by non-linear curve fit analysis using Prism software (GraphPad, San Diego, USA) with R2 > 0.87 and P (run tests) as parameter of goodness-of-fit.
