# Supplementary material for: The BH3 mimetic (±) gossypol induces ROS-independent apoptosis and mitochondrial dysfunction in human A375 melanoma cells in vitro
Source: Arch Toxicol. 2021 Feb 1;95(4):1349–65. doi: 10.1007/s00204-021-02987-4 (PMC8032633; doi:10.1007/s00204-021-02987-4)
Supplement: Supplementary file 2 — Supplementary file2 (DOCX 137 KB) [file 204_2021_2987_MOESM2_ESM.docx]

# **The BH3 mimetic** **(±) gossypol induces ROS-independent apoptosis and mitochondrial dysfunction in human A375 melanoma cells *in vitro***

Lisa Haasler*^1^, Arun Kumar Kondadi^1^, Thanos Tsigaras^1^, Claudia von Montfort^1^, Peter Graf^1^, Wilhelm Stahl^1^, Peter Brenneisen^1^

^1^Institute of Biochemistry and Molecular Biology I, Medical Faculty, Heinrich Heine University Düsseldorf, Düsseldorf, Germany

*Corresponding author, e-mail: lisa.scharf@hhu.de


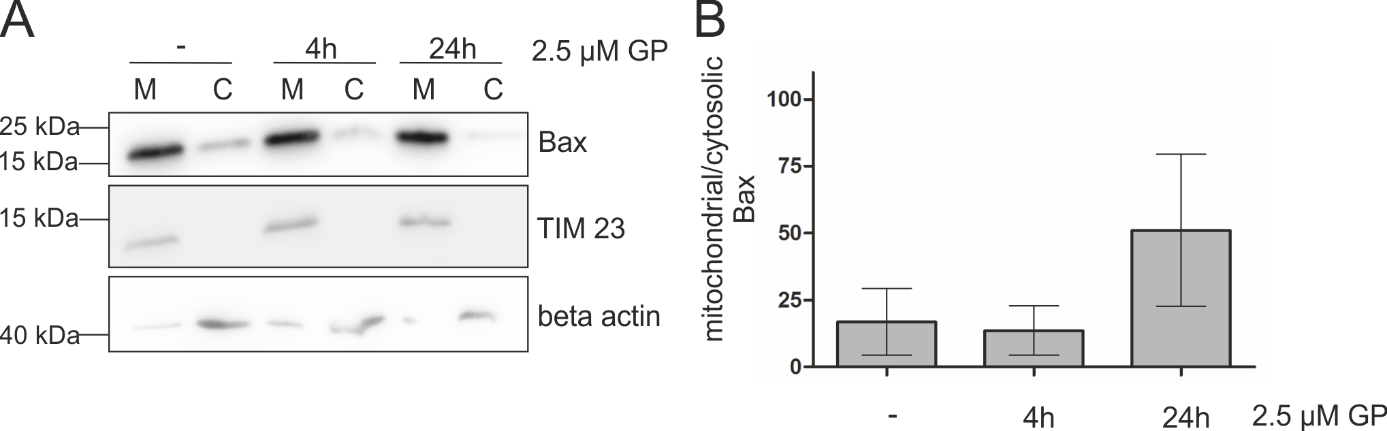


**Supplemental figure 2:** GP treatment led to a shift on Bax localization towards mitochondria. **a** After incubation with 2.5 µM GP for 4 and 24h, respectively, cells were lysed and separated in cytosolic and mitochondrial fraction with subsequent western blotting analysis of Bax. TIM 23 served as loading control for the mitochondrial and beta actin for the cytosolic fraction. **b** Quantification of the ratio between mitochondrial and cytosolic localized Bax by a densitometric analysis using FusionCapt Advance software. Data represent means ± S.E.M of three independent experiments.
