# Supplementary material for: The BH3 mimetic (±) gossypol induces ROS-independent apoptosis and mitochondrial dysfunction in human A375 melanoma cells in vitro
Source: Arch Toxicol. 2021 Feb 1;95(4):1349–65. doi: 10.1007/s00204-021-02987-4 (PMC8032633; doi:10.1007/s00204-021-02987-4)
Supplement: Supplementary file 3 — Supplementary file3 (DOCX 100 KB) [file 204_2021_2987_MOESM3_ESM.docx]

# **The BH3 mimetic** **(±) gossypol induces ROS-independent apoptosis and mitochondrial dysfunction in human A375 melanoma cells *in vitro***

Lisa Haasler*^1^, Arun Kumar Kondadi^1^, Thanos Tsigaras^1^, Claudia von Montfort^1^, Peter Graf^1^, Wilhelm Stahl^1^, Peter Brenneisen^1^

^1^Institute of Biochemistry and Molecular Biology I, Medical Faculty, Heinrich Heine University Düsseldorf, Düsseldorf, Germany

*Corresponding author, e-mail: lisa.scharf@hhu.de


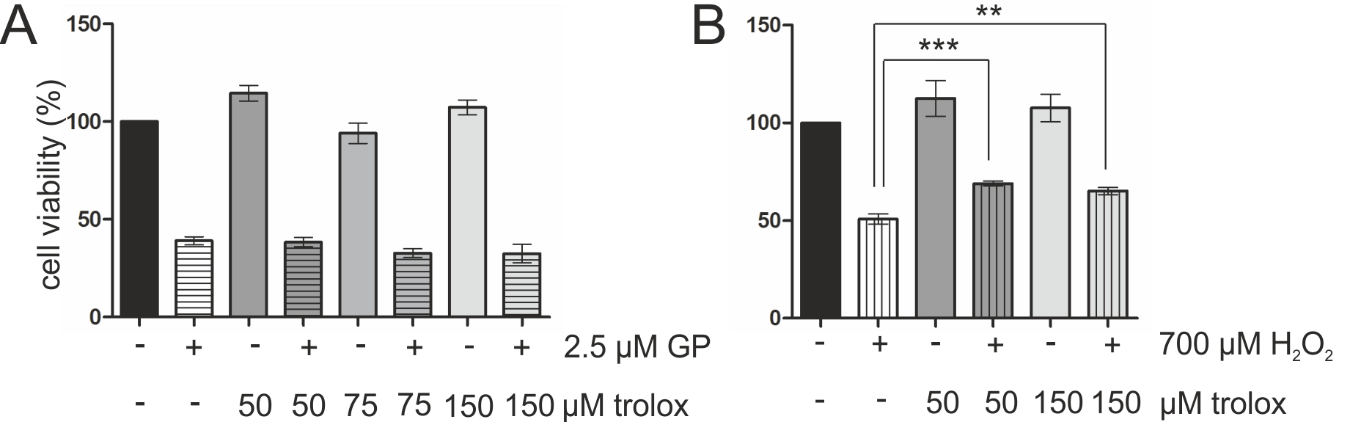


**Supplemental figure 3:** Effect of GP on ROS generation in A375 melanoma cells in combination with the antioxidant Trolox. a To show a ROS independent mechanism of GP, in addition to the NAC experiments (Fig. 6d,e), A375 melanoma were pretreated with 50, 75 and 150 µM of the antioxidant (±)-6-Hydroxy-2,5,7,8-tetramethylchromane-2-carboxylic acid (trolox) for 4h and subsequently, 2.5 µM GP (a) or 700 µM H2O2 as positive control (b), respectively, was added for further 24 h. The cell viability was measured by MTT assay and the mock-treated control was set at 100%. Data represents means S.E.M., n=3-4. One-way ANOVA with Dunnett’s Multiple Comparison Test was used for the determination of statistical significance between H_2_O_2_ and H_2_O_2_ + Trolox. **p<0.01, ***p<0.001
