# Supplementary material for: The BH3 mimetic (±) gossypol induces ROS-independent apoptosis and mitochondrial dysfunction in human A375 melanoma cells in vitro
Source: Arch Toxicol. 2021 Feb 1;95(4):1349–65. doi: 10.1007/s00204-021-02987-4 (PMC8032633; doi:10.1007/s00204-021-02987-4)
Supplement: Supplementary file 4 — Supplementary file4 (DOCX 202 KB) [file 204_2021_2987_MOESM4_ESM.docx]

# **The BH3 mimetic** **(±) gossypol induces ROS-independent apoptosis and mitochondrial dysfunction in human A375 melanoma cells *in vitro***

Lisa Haasler*^1^, Arun Kumar Kondadi^1^, Thanos Tsigaras^1^, Claudia von Montfort^1^, Peter Graf^1^, Wilhelm Stahl^1^, Peter Brenneisen^1^

^1^Institute of Biochemistry and Molecular Biology I, Medical Faculty, Heinrich Heine University Düsseldorf, Düsseldorf, Germany

*Corresponding author, e-mail: lisa.scharf@hhu.de


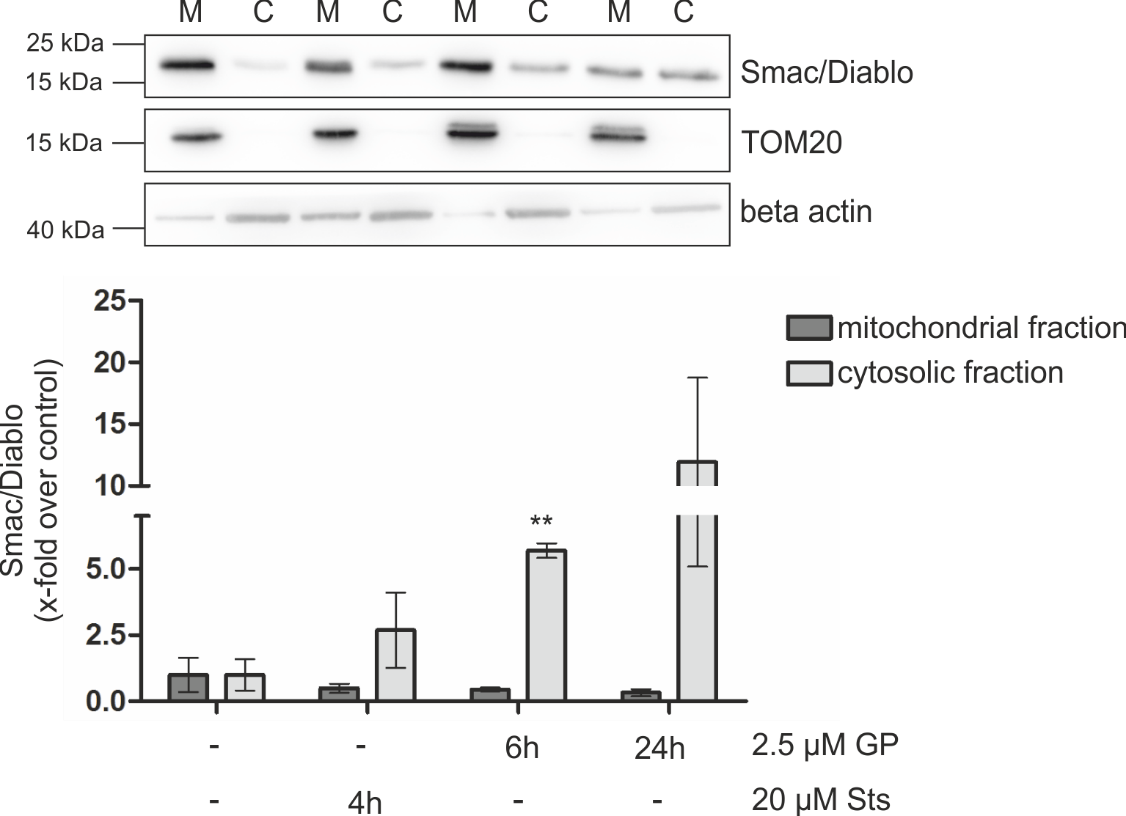


**Supplemental figure 4:**  Release of Smac/DIABLO into the cytosol in A375 melanoma after GP treatment. After incubation with 2.5 µM for 6 and 24 h, respectively, or with 20 µM staurosporine (sts) for 4 h as positive control, cells were lysed and separated in mitochondrial and cytosolic fraction with following western blot analysis. Beta-actin served as loading control for the cytosolic fraction and TOM20 for the mitochondrial fraction. Densitometry was done by using FusionCapt Advance software. Mock-treated control of both mitochondrial and cytosolic fraction was set to 1. Data are means ± S.E.M., n=3
